# Quantitative lung ultrasound to understand pathophysiology and guide surfactant therapy in neonates with meconium aspiration

**DOI:** 10.1016/j.jped.2026.101550

**Published:** 2026-04-27

**Authors:** Daniele De Luca

**Affiliations:** a Division of Paediatrics, Transportation and Neonatal Critical Care, “A.Beclere” Medical Centre, APHP-Paris Saclay University, Paris, France; b Pathophysiology and Therapeutic Innovation Unit, INSERM U-999, Paris Saclay University, Paris, France

In this number of the *Jornal de Pediatria*, Dr. Chen and coworkers publish an interesting multicenter, prospective, diagnostic accuracy, cohort study to guide surfactant therapy in neonates with meconium aspiration syndrome (MAS) [1]. The study adds to our knowledge and provides some important novelties. For the first time, the authors demonstrated that quantitative lung ultrasound (LUS) is a useful and accurate method of guiding bolus surfactant administration in neonates with MAS. MAS is a severe respiratory disorder that may trigger neonatal acute respiratory distress syndrome (NARDS) and is linked to high mortality rates [2]. MAS is also a relatively rare condition, thus it is difficult to conduct high-quality studies to improve its clinical management, and the authors should be commended for this.

Surfactant is a cornerstone of neonatal critical care and represents the main treatment for the respiratory distress due to primary surfactant deficiency (i.e. hyaline membrane disease most commonly occurring in preterm infants). MAS and meconium-induced NARDS are characterized by a complex severe qualitative/quantitative surfactant dysfunction [[3], [4], [5]] and clinical data show that surfactant bolus improves oxygenation, decreases the need for extra-corporeal life support and shortens the duration of invasive ventilation and hospital stay [6,7]. Although surfactant is not licensed for indications other than hyaline membrane disease, it is often used in MAS patients and an estimation based on the birth rate, the prevalence of MAS and a mean birth weight of 3500 g yields to approximately 18,000 and 90,000 poractant-alpha vials being used every year in France and United States, respectively [8]. The estimation was done considering 200 mg/Kg dose which is the optimal dose for preterm neonates with hyaline membrane disease [9,10], but the best dose to use in MAS, as in any NARDS patient, is far from being clarified as exogenous surfactant is likely inactivated by secretory phospholipase A2 which is significantly active in the lung tissue of MAS patients [11,12].

This background makes it even more important and urgent to understand the pathophysiology of patients with MAS and to guide therapy for those who may require surfactant treatment. This is clinically relevant because not all cases of MAS progress to NARDS and require surfactant; milder cases can be safely and effectively treated with continuous positive airway pressure [13]. LUS constitutes a reliable tool to understand the pathophysiology in terms of lung aeration, that is the amount of lung volume available for gas exchange, as already demonstrated in acute respiratory distress in adults and in several other neonatal respiratory disorders [14].

Chen et al. demonstrate the extremely high reliability of the LUS aeration score in predicting the need for surfactant in patients with MAS, where this outcome was identified by worsening oxygenation impairment, fulfilling the criterion of at least moderate NARDS [15]. This high diagnostic accuracy is consistent in every subgroup analysis and remains significant upon multivariate adjustment. This elegantly designed diagnostic accuracy study follows STARD guidelines and presents several points of strength such as:

- Multicenter design.
- Large sample size calculated on a very high diagnostic accuracy target (area under the curve (AUC) = 0.95).
- Concealment ensuring that physicians in charge did not use LUS score to decide the surfactant therapy.
- Detailed and strict statistical methodology.

In addition to the main results, these features also enabled the high inter-rater reliability of LUS aeration score calculation to be confirmed, as demonstrated in previous studies, and the consistency of the findings with those observed in neonates with other respiratory disorders [14].

It should be noted that this study is not a randomized controlled trial, but rather a high-quality diagnostic accuracy study. It was conducted and reported in accordance with the dedicated STARD guidelines [16]. This is the correct design for evaluating a diagnostic or monitoring tool. The importance of trials must not distract us from the fact that diagnostics are not therapeutics and cannot change outcomes in themselves. They must be used and correctly interpreted to improve our understanding of patient pathophysiology and adapt the subsequent therapy. Unfortunately, the study also suffers from some methodological issues that raise doubts and leave open questions for future research projects.

The main problem is that the study uses a suboptimal LUS aeration score which does not scan the posterior lung zones. This is a fatal flaw since these are the dependent lung areas whose aeration and perfusion is significantly influenced by the gravity [17]. More and above this, MAS is known to be a heterogeneous lung disorder and lung aeration heterogeneity may go unnoticed or underestimated without a comprehensive evaluation of all lung zones [18]. This has the potential to have severe consequences, as heterogeneous lungs are at a higher risk of lung stress and ventilator-induced lung injury, which can lead to an outcome that is worse than the original severity of the MAS [19]. The exploration of posterior lung zones to calculate a specific extended LUS aeration score is easy [20], does not require patient pronation and is strongly recommended by the European guidelines for quantitative LUS [14].

The results presented by Chen et al. indicate a score threshold of 6 for surfactant administration, which seems fairly low. This is because the simplified score was used rather than the extended score, potentially missing the presence of large consolidations, which would significantly increase the score. In fact, the universally accepted threshold to guide surfactant replacement is 8 in neonates with hyaline membrane disease, which is a homogeneous disorder [21].

Finally, authors did not describe how many patients evolve towards NARDS, and particularly moderate-severe NARDS. This is clinically relevant because MAS is a frequent NARDS trigger [15], and the diagnostic accuracy of LUS would be even more useful to identify the patients with more severe evolution. In fact, surfactant efficacy is generally higher with earlier administration [8], and the most important outcomes that surfactant can influence, such as oxygenation improvement and avoidance of extracorporeal life support, are easily detectable only in the most severe cases [6,7]. Therefore, the diagnostic accuracy of LUS in the most severe patients would provide valuable clinical information that goes beyond simply understanding patient pathophysiology. Anyhow, understanding patient pathophysiology and monitor its evolution is pivotal for a more personalized respiratory care and this would otherwise be unavailable without LUS, unless other techniques were used (e.g. electrical impedance tomography or computerized scan).

In conclusion, Chen et al. provide useful insights into a rare and life-threatening neonatal disorder. They clearly demonstrate that LUS is essential for understanding patient pathophysiology, guide surfactant administration and hence providing a more personalized neonatal critical care medicine.

However, given the limitations of their study, further steps are needed to fully exploit the potential of LUS in these patients.

## Data availability

N/A.

## Conflicts of interest

The author declares no conflicts of interest.
